# Myocardial Pyruvate Metabolism Before and After CABG in Three-Vessel Disease Using Hyperpolarized Carbon-13 MRI

**DOI:** 10.21203/rs.3.rs-9570351/v1

**Published:** 2026-05-13

**Authors:** Gaurav Sharma, Jaidip Jagtap, Sung-Han Lin, Jae Mo Park, Crystal Harrison, Jennine Leary, Sarah McNeil, Corey Mozingo, Craig Malloy, Matthias Peltz, Michael Jessen

**Affiliations:** The University of Texas Southwestern Medical Center; Mayo Clinic; The University of Texas Southwestern Medical Center; The University of Texas Southwestern Medical Center; The University of Texas Southwestern Medical Center; The University of Texas Southwestern Medical Center; The University of Texas Southwestern Medical Center; The University of Texas Southwestern Medical Center; The University of Texas Southwestern Medical Center; UT Southwestern Medical Center; The University of Texas Southwestern Medical Center

## Abstract

Hyperpolarized [1-^13^C]pyruvate MRI provides real-time, radiation-free insight into myocardial pyruvate metabolism and mitochondrial oxidative capacity, yet its utility in patients with severe multivessel coronary artery disease before and after surgical revascularization remains unknown. Here we show, in the first prospective study applying this technique to three-vessel disease, that serial imaging is feasible and safe with no serious adverse events in 22 injections. Healthy myocardium displayed a bicarbonate-to-total hyperpolarized carbon product ratio of 0.66 ± 0.13, indicating that the majority of pyruvate undergoes oxidation. Patients exhibited significantly lower ratios in the inferolateral segment (P = 0.03) with reciprocal increases in lactate, and global ratios correlated with left ventricular ejection fraction (r = 0.695, P = 0.006). Post-revascularization changes were heterogeneous across individuals. These findings establish hyperpolarized 13C MRI as a powerful tool for noninvasive assessment of myocardial metabolism in multivessel coronary artery disease and support its potential for longitudinal therapeutic monitoring.

## INTRODUCTION

Coronary artery bypass grafting (CABG) remains the standard of care for patients with severe multivessel coronary artery disease (CAD), particularly those with left main or three-vessel involvement accompanied by reduced left ventricular function^1,2^. The success of surgical revascularization depends critically on the presence of viable myocardium capable of functional recovery following restoration of blood flow ^3,4^. Current methods for assessing ischemic burden or myocardial viability include dobutamine echocardiography, myocardial perfusion imaging, delayed enhancement cardiac magnetic resonance imaging (MRI), and ^18^F-fluorodeoxyglucose positron emission tomography, which evaluates glucose metabolic activity^3,5–8^. Although these modalities provide complementary information, they assess metabolic status indirectly or require ionizing radiation which limits serial studies.

Hyperpolarized ^13^C MRI is an emerging noninvasive method that provides unprecedented real-time metabolic insight into mitochondrial function in human patients, entirely free of ionizing radiation.^9–12^ Intravenous injection of hyperpolarized [1-^13^C]pyruvate enables real-time visualization of myocardial pyruvate metabolism, where flux through pyruvate dehydrogenase (PDH) to bicarbonate reflects mitochondrial oxidative capacity, and conversion via lactate dehydrogenase (LDH) to lactate, indicates redox state and overall carbohydrate metabolism.^13,14^ In ischemic myocardium, reduced oxygen delivery impairs mitochondrial oxidative phosphorylation, diminishing fatty acid oxidation and PDH-mediated pyruvate oxidation while increasing reliance on anaerobic glycolysis. These metabolic shifts may precede structural changes detectable by conventional imaging. Detecting PDH flux, a unique feature of mitochondrial function, may better identify patients likely to benefit from revascularization. The ability to assess apparent PDH activity using HP [1-^13^C]pyruvate MRI therefore offers a unique window into myocardial metabolic status that may inform ischemia and viability assessment and improve therapeutic decision-making.

The objectives of this study were threefold: to characterize myocardial pyruvate metabolism using HP [1-^13^C]pyruvate MRI in patients with three-vessel coronary artery disease compared with healthy controls; to evaluate regional metabolic alterations corresponding to coronary artery territories; and to assess metabolic changes following coronary artery bypass grafting. The findings establish feasibility for this metabolic imaging approach in the longitudinal evaluation of patients undergoing surgical revascularization and provide preliminary evidence for its potential role in assessing myocardial metabolism in real-time to guide management of ischemic heart disease.

## RESULTS

### Study Population Characteristics

Baseline characteristics of the study population are presented in Table 1. Healthy controls (n=9) were younger (36 ± 4 versus 64 ± 12 years, p<0.001), more likely female (66.7% versus 0%), and had lower body mass index (24.2 ± 1.0 versus 29.1 ± 1.2 kg/m^2^, p<0.01) compared with CAD patients (n=5). By design, healthy controls had no cardiovascular comorbidities, whereas CAD patients demonstrated a high burden of traditional cardiovascular risk factors including hypertension (80%), hyperlipidemia (60%), former tobacco use (60%), and diabetes or prediabetes (40%). Two patients (40%) had obstructive sleep apnea and two (40%) had a history of malignancy. All CAD patients had angiographically confirmed three-vessel coronary artery disease with significant stenoses requiring elective surgical revascularization (Supplementary Table 2 and Supplementary Methods). Pre-CABG imaging was performed 14 ± 9 days before surgery, and post-CABG imaging occurred 120 ± 23 days following the procedure.

### Safety and Feasibility of Hyperpolarized ^13^C MRI

All 22 hyperpolarized [1-^13^C]pyruvate injections (10 healthy + 6 pre-CABG + 6 post-CABG) were completed without serious adverse events. No subjects experienced clinically significant hemodynamic perturbations, arrhythmias, allergic reactions, or injection site reactions during the study or during the 2-hour follow up. Side effects were limited to mild lip flushing or a metallic taste immediately following injection, resolving within seconds. One healthy volunteer (H7) and one CAD patient (Pre-CABG5) were excluded from analysis due to technical failures, yielding a per-protocol technical success rate of 87.5% (14/16 acquisitions). Quality control parameters for all successful acquisitions were within predefined acceptable ranges (Figure 2B), including temperature (28.0 ± 2.6°C), pH (7.75 ± 0.19), pyruvate concentration (245 ± 11 mM), polarization signal (4500 ± 1483 a.u.), time from dissolution to injection (48 ± 8 seconds), and residual radical concentration (1.61 ± 1.60 μM, all within optimal range). In selected subjects, a second HP [1-^13^C]pyruvate injection was performed at a different imaging axis (long-axis versus short-axis) within the same session. Corresponding short-axis and long-axis metabolite maps confirmed consistent spatial localization of pyruvate, bicarbonate, and lactate signals across the 17-segment model (Supplementary Figure 3).

### Regional Metabolic Assessment: Healthy Controls Versus Pre-CABG

Representative dynamic image series from a healthy volunteer (Figure 2C, upper rows) and a pre-CABG patient (Figure 2E) capture the metabolic bolus transit and its characteristic differences between normal and ischemic myocardium. Extended temporal resolution of this transit is detailed in Supplementary Figure 4. The high-signal pyruvate bolus is first visualized in the left ventricular cavity (reflecting the 25-second delay in data acquisition after injection), while signal appearance in the right ventricular cavity is observed subsequently during recirculation. Representative normalized dynamic curves demonstrate robust oxidative metabolic activity in a healthy volunteer (Figure 2D), characterized by a dominant bicarbonate peak (AUC_Bic_ = 13.54 a.u.). Conversely, the pre-CABG patient (Figure 2F) exhibits suppressed bicarbonate production (AUC_Bic_ = 5.93 a.u.) and a flattened, delayed metabolic conversion profile compared to the sharp, early peak observed in the healthy control (Figure 2D), despite an identical injection protocol. Representative Bic/THCP ratio maps for each individual subject are shown in Figure 3A–B.

In healthy controls, Bic/THCP ratios were relatively uniform across all six mid-ventricular myocardial segments (mean of all signal from the LV myocardium, 0.66 ± 0.13; Figure 5A), indicating homogeneous PDH-mediated metabolic activity. In contrast, pre-CABG patients demonstrated marked regional metabolic heterogeneity with reduced Bic/THCP ratios (mean global ratio 0.51 ± 0.18; Figure 5A), most notably in the inferolateral territories (p=0.03). Although all patients had 3-vessel CAD, this cohort had a high prevalence of severe circumflex and right CAD (Supplementary Table 2). Segment-by-segment comparison revealed significantly reduced bicarbonate production in the inferolateral segment (AHA segment 11) of pre-CABG patients compared with healthy controls (p=0.03; Figure 3H), corresponding to the circumflex or right coronary artery territory. Representative angiographic images (Figure 3C) illustrate the lesion distribution in one patient. Other segments showed non-significant trends toward reduced Bic/THCP ratios in the pre-CABG group, including the inferior segment (p=0.06; Figure 3G). Because Bic/THCP and Lac/THCP sum to 1 by definition, reduced Bic/THCP necessarily corresponds to elevated Lac/THCP at both the global and voxel level. The spatial distribution of Lac/THCP across individual voxels (Supplementary Figure 5) provides a complementary view of regional metabolic heterogeneity, with higher values in territories corresponding to stenotic vessels. As expected from the Bic/THCP results, the inferolateral segment (segment 11) showed significantly elevated Lac/THCP in pre-CABG patients versus healthy controls (p=0.03; Supplementary Figure 5G). Anatomical overlay of individual metabolite maps onto proton images confirmed accurate cardiac localization of the hyperpolarized ^13^C signal and illustrated the regional differences in bicarbonate distribution between healthy and ischemic myocardium (Supplementary Figure 7). Mid-ventricular polar maps (Figure 5A and 5B) summarize the AHA 17-segment distribution of Bic/THCP ratios and Lac/THCP ratios across all three groups. These maps highlight the regional metabolic impairment concentrated in the inferolateral and inferior segments of pre-CABG patients, contrasting with the relatively uniform metabolic pattern observed in healthy controls.

### Ventricular Function

Pre-CABG patients exhibited localized regional metabolic impairments against a background of otherwise intact global oxidative activity, as shown in Figure 3A–3I. Across the combined cohort of healthy controls and pre-CABG patients, mean global Bic/THCP ratio correlated significantly with left ventricular ejection fraction (Spearman r=0.695, p=0.006; Figure 3L), demonstrating that impaired PDH-mediated pyruvate oxidation is significantly associated with reduced systolic function. Functional assessment confirmed that left ventricular ejection fraction (LVEF) was significantly lower in pre-CABG patients compared with healthy controls (43.0 ± 10.5% versus 60.2 ± 7.8%; p=0.02; Figure 3K). Pre-CABG patients demonstrated reduced systolic function with wall motion abnormalities, consistent with their underlying three-vessel disease. All patients were clinically stable and on guideline-directed medical therapy at the time of imaging. Heart rate did not differ significantly between groups (p=0.55; Figure 3J), nor did left ventricular stroke volume (p=1.00; Figure 3M) or cardiac output (p=1.00; Figure 3N). Right ventricular ejection fraction (p=0.61; Figure 3O) and right ventricular cardiac output (p=0.80; Figure 3P) were also comparable between groups. Following CABG, there was no difference in ejection fraction compared with pre-CABG values (43.0 ± 10.5% pre-CABG versus 44.8 ± 10.2% post-CABG; p=0.44; Figure 4J), Other hemodynamic parameters including heart rate, stroke volume, and cardiac output remained stable following surgery (Figure 4I, 4L–O).

### Metabolic Changes Following CABG

Paired pre-CABG and post-CABG Bic/THCP ratio maps for all five patients are presented in Figure 4A–B. Visual inspection of individual patient maps revealed heterogeneous responses to surgical revascularization. Notably, Post-CABG3 demonstrated a marked decrease in Bic/THCP ratios compared with Pre-CABG3, while Post-CABG6 showed improvement relative to Pre-CABG6, illustrating the interpatient variability inherent to this small cohort. In aggregate, the mean global Bic/THCP ratio did not change significantly following CABG (pre-CABG: 0.51 ± 0.18 versus post-CABG: 0.42 ± 0.34; mean δ: −0.09 ± 0.27; Figure 5A). Regional quantitative comparisons demonstrated no statistically significant changes in any individual segment (Figure 4C–H), including the inferolateral segment (p=1.00; Figure 4G). However, Bic/THCP ratios (Figure 5A) revealed trends toward improvement in specific segments, with mean normalized ratios increasing from 0.49 ± 0.18 (pre-CABG) to 0.57 ± 0.32 (post-CABG; mean δ: +0.09 ± 0.27).

Corresponding Lac/THCP ratio maps in CAD patients showed reciprocal trends to the Bic/THCP ratio, with heterogeneous responses in lactate production observed in previously ischemic territories following revascularization (Supplementary Figure 6). While this study was not primarily designed to determine kinetic rate constants, we explored the feasibility of mapping apparent kinetic rate constants with spatial distributions mapping the conversion of pyruvate conversion (Supplementary Figures 8-10). Spatial distributions of *k_PB_* (pyruvate-to-bicarbonate; Supplementary Figure 8) revealed heterogeneous post-CABG changes, while *k_PL_* (pyruvate-to-lactate; Supplementary Figure 9) remained relatively stable following revascularization, suggesting that metabolic recovery, where present, primarily involves restoration of oxidative capacity rather than cytosolic redox state. Corresponding polar maps are shown in Supplementary Figure 10. A trend toward a positive correlation between mean global Bic/THCP ratio and LV ejection fraction was observed in the pre- and post-CABG cohort (Spearman r = 0.575, p = 0.082; Figure 4K), consistent with the strong relationship observed in the combined healthy and pre-CABG analysis, though it did not reach statistical significance in this smaller paired group.

### Circulating Metabolic Substrates

Results from serum metabolite analysis are shown in Figures 6A–D. As anticipated, triglyceride levels were significantly elevated in pre-CABG patients compared with healthy controls (p=0.011; Figure 6B). Serum pyruvate, prior to the HP exam, was increased following CABG, perhaps indicating diet or medications changes (p=0.002; Figure 6C). Insulin levels showed a non-significant trend toward reduction following CABG (Figure 6A), while non-esterified fatty acid levels remained stable across all groups (Figure 6D). Medication changes following CABG were documented (Figure 6E). All five CAD patients received lipid-lowering agents and antiplatelet therapy at both time-points. Diuretic use increased from one to five patients post-CABG, likely due to post-operative fluid management protocols, while SGLT2 inhibitors were initiated in two patients. Calcium channel blocker use decreased from four to two patients, and nitrate use decreased from four to one patient following revascularization.

## DISCUSSION

This is the first report of imaging the products of HP [1-^13^C]pyruvate in patients with documented three-vessel coronary artery disease before and after surgical revascularization. This study demonstrates the feasibility and safety of HP [1-^13^C]pyruvate MRI for serial evaluation of mitochondrial function in the heart of patients with severe coronary artery disease and abnormal ventricular function. The principal findings include: high Bic/THCP in healthy myocardium, a correlation between global metabolic activity and ventricular function across all subjects, and a variable metabolic response following CABG. In selected subjects there were regional reductions in PDH-mediated bicarbonate production in territories corresponding to severe coronary stenoses. These observations establish a foundation for larger studies examining the role of ^13^C metabolic imaging in assessment of patients with ischemic heart disease.

Earlier spectroscopy studies of pyruvate metabolism in human myocardium found that only a small fraction of the injected pyruvate was oxidized in mitochondria and the majority was reduced to lactate. Bic/THCP ranged from 0.07 to 0.23 in healthy subjects or in otherwise-healthy women prior to therapy for breast cancer^10 15^. The current finding that Bic/THCP was much higher (0.66 in healthy myocardium and 0.51 even in patients with severe coronary artery disease), indicates that in stable subjects the majority of injected pyruvate undergoes oxidative metabolism. This difference between current results and earlier reports is due to acquisition of HP[1-^13^C]lactate signal from the blood pool in the right and left ventricular cavities in spectroscopy studies, thus reducing the ratio Bic/(Bic+Lac). The current results demonstrate that ^13^C imaging is essential for exams in patients. Under conditions of persistent oxygen limitation, the myocardium becomes more reduced, leading to inactivation of the PDH complex and impaired decarboxylation of pyruvate. Metabolism of pyruvate and other carbohydrates shifts to lactate production because of the high NADH/NAD^+^ in ischemic muscle. Overall glucose metabolism shifts to anaerobic glycolysis. In the current study, all patients had angiographically significant three vessel CAD and all subjects were clinically stable at the time of the exam without complaints of angina and presumably without ischemia. However, in this population, the reduction in bicarbonate production observed in the inferolateral segment (AHA segment 11, p=0.03) correlated with the angiographic severity of individual stenoses in the right coronary artery or the circumflex system, perhaps indicating subclinical ischemia.

When all subjects, healthy and pre-CABG patients were grouped, there was a correlation (r=0.695, p=0.006) between PDH-mediated oxidative capacity (Bic/THCP ratio) and LVEF. As suggested, this effect could be due to subclinical ischemia. However, PDH flux is very sensitive to competing substrates. Increased oxidation of long-chain fatty acids or ketones may substantially suppress PDH flux even in normal myocardium^13,14^, and it is conceivable that some drug therapies such as beta adrenergic blockers may impact PDH flux. The possibility that systemic changes in oxidizable substrate concentrations must be considered is supported by the findings of small differences in the concentrations of triglycerides, fatty acids and other substrates in subsets. Thus, although the current results are consistent with an effect of severe CAD on both left ventricular function and pyruvate oxidation, further work on the effects of competing substrates and drug effects is essential to determine if reduced pyruvate oxidation indicates subclinical and perhaps reversible ischemia.

The metabolic response to surgical revascularization observed in this study was heterogeneous across patients and segments. While the normalized Bic/THCP ratio showed a numerical increase from 0.49 ± 0.18 to 0.57 ± 0.32 (δ: +0.09 ± 0.27; Figure 5B), the unnormalized global ratio exhibited a slight decrease (0.51 ± 0.18 to 0.42 ± 0.34; δ: −0.09 ± 0.27; Figure 5A), and individual segment comparisons did not reach statistical significance (Figure 4C–H). This discordance likely reflects the substantial inter-patient variability inherent to a five-patient cohort, where individual outliers (such as Post-CABG3, who demonstrated markedly reduced metabolic activity) can disproportionately influence group-level statistics. The wide standard deviations observed in δ values across all segments (Figure 5A–B and Supplementary Figure 10) underscore this challenge. Despite the absence of statistically significant paired changes, the direction of metabolic recovery in several individual patients aligns with the expected physiological response to restored coronary blood flow, namely improved oxygen delivery and reactivation of mitochondrial oxidative phosphorylation. Serial perfusion-metabolism studies using PET have demonstrated that recovery of glucose metabolism may precede or parallel functional improvement following revascularization^16^, and the 120-day post-CABG time point in the present study may capture a period of ongoing metabolic remodeling where resting oxidative metabolic activity has not yet fully normalized.

Current clinical practice for myocardial viability assessment relies primarily on delayed enhancement cardiac MRI and ^18^F-FDG PET^6^. Delayed enhancement imaging identifies irreversible scar based on gadolinium kinetics but cannot directly assess metabolic activity in non-enhanced segments. Monitoring carbohydrate metabolism using [^18^F]fluoro-deoxyglucose is thought to be the most sensitive method for identifying heart muscle that will recover after revascularization^6,17^. However, a clear benefit for FDG PET-assisted care was not demonstrated in one study^18^, suggesting that alternative methods may provide additional information. Surprisingly, the impact of bypass surgery on myocardial carbohydrate metabolism has not been extensively evaluated. Glucose metabolism may be reduced by CABG, consistent with results in some of the current patients ^19,20^. Hyperpolarized [1-^13^C]pyruvate MRI offers several potential advantages for myocardial metabolic assessment. The technique directly visualizes metabolic conversion rather than static tracer accumulation, providing mechanistic information about PDH activity that underlies cellular energy production. The absence of ionizing radiation permits serial imaging to monitor therapeutic response. Furthermore, integration with anatomical proton MRI within a single examination enables comprehensive characterization of structure, function, and metabolism. Future studies directly comparing HP [1-^13^C]pyruvate MRI with established stress test and viability methods are warranted to define the incremental value of metabolic imaging.

The small sample size limits statistical power and precludes definitive conclusions regarding metabolic differences and treatment effects. The substantial age and sex differences between healthy controls and CAD patients represent potential confounders. Although patients were clinically stable at the time of the study, there was no independent assessment of possible ischemia at the time of the exam. Further, the hemodynamic significance of each lesion was not assessed by invasive methods, so even moderately significant stenosis could have been associated with downstream ischemia. The lack of direct comparison with established viability methods (^18^F-FDG PET, delayed enhancement MRI) prevents assessment of incremental diagnostic value.

The findings of this study have several potential clinical implications that warrant validation in appropriately powered cohorts. First, pre-operative metabolic imaging with HP [1-^13^C]pyruvate MRI may inform patient selection for surgical revascularization by identifying ischemic myocardium. The significant correlation between Bic/THCP ratio and LVEF observed in this study (r=0.695, p=0.006) suggests that metabolic imaging provides complementary prognostic information that could enhance existing decision-making frameworks in low ejection fraction patients. Second, serial metabolic imaging may enable non-invasive monitoring of therapeutic response, not only to revascularization but also to emerging metabolic modulators ^21–23^. Third, integration of metabolic imaging with first-pass perfusion assessment within a single MRI examination may enable characterization of perfusion-metabolism mismatch analogous to PET-based approaches, potentially identifying hibernating myocardium with greater specificity and without the logistical burden of coordinating two separate imaging modalities. Finally, the current study demonstrated the feasibility of collecting kinetic rate constant data from all myocardial segments. Such data have been used in earlier studies to calculate the apparent rate constants for pyruvate conversion to bicarbonate or lactate. The current pulse sequence with a delay of 25 seconds prior to data acquisition is not suitable for the earlier mathematical models, but it would be a simple matter to collect appropriate data. Development of alternative hyperpolarized substrates, such as [2-^13^C]pyruvate for Krebs cycle assessment or [^13^C]acetoacetate for ketone bodies may provide additional metabolic dimensions relevant to ischemic heart disease ^24,25^.

This study demonstrates the feasibility and safety of HP [1-^13^C]pyruvate MRI for assessing myocardial pyruvate metabolism in patients with three-vessel coronary artery disease before and after CABG. Regional metabolic impairment corresponding to coronary territories and significant correlations with ventricular function support the potential of this technique as a non-invasive metabolic biomarker. These findings establish a foundation for larger validation studies examining the role of metabolic imaging in myocardial viability assessment and therapeutic monitoring.

## METHODS

### Study Design and Population

**Figure 1:**
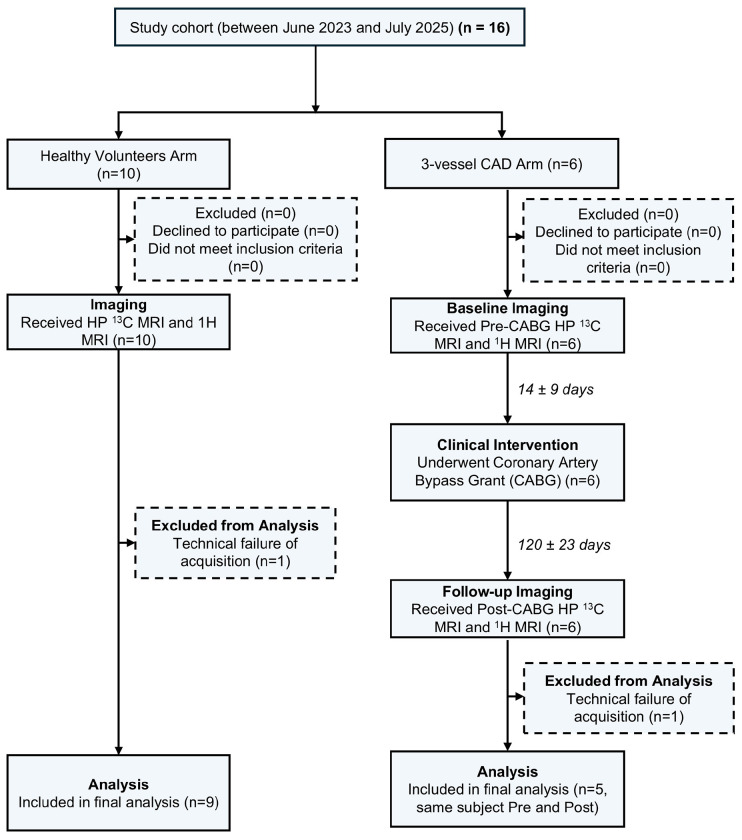
Study Flow Diagram. Sixteen subjects were enrolled between June 2023 and July 2025, comprising 10 healthy volunteers and 6 patients with three-vessel coronary artery disease (CAD). One subject from each cohort was excluded due to technical acquisition failures, yielding final cohorts of 9 healthy controls and 5 CAD patients. CAD patients underwent hyperpolarized (HP) ^13^C MRI at two time points: before coronary artery bypass grafting (CABG) (14 ± 9 days pre-surgery) and after CABG (120 ± 23 dayspost-surgery). ^1^H = proton.

This prospective, single-center study enrolled participants between June 2023 and July 2025 at UT Southwestern Medical Center (Figure 1). The study protocol was approved by the institutional review board (IRB No. STU-2020-0953, investigational new drug application no. 133229, NCT06047028), and all participants provided written informed consent. The study population comprised two cohorts: healthy volunteers without cardiovascular disease or risk factors, and patients with angiographically confirmed three-vessel coronary artery disease scheduled for elective CABG.

The detailed enrollment criteria are available in Supplementary Methods. Briefly, the inclusion criteria for healthy volunteers included age 18-80 years, absence of cardiovascular disease or major cardiovascular risk factors (hypertension, diabetes mellitus, hyperlipidemia, tobacco use), body mass index less than 30 kg/m^2^, and no contraindications to MRI. Inclusion criteria for the CAD cohort included age 18 years or older, angiographically documented three-vessel CAD with planned CABG, and clinical stability allowing pre-operative imaging. Exclusion criteria for both groups included estimated glomerular filtration rate less than 60 mL/min/1.73m^2^, contraindications to MRI, pregnancy or lactation, and inability to provide informed consent. Of 16 enrolled subjects, 10 were healthy volunteers and 6 were CAD patients. One healthy volunteer and one CAD patient were excluded due to technical acquisition failures, yielding final cohorts of 9 healthy controls and 5 CAD patients for analysis. CAD patients underwent imaging at two time points: pre-CABG (14 ± 9 days before surgery) and post-CABG (120 ± 23 days after surgery).

### Hyperpolarized [1-^13^C]Pyruvate Preparation and Quality Control

**Figure 2:**
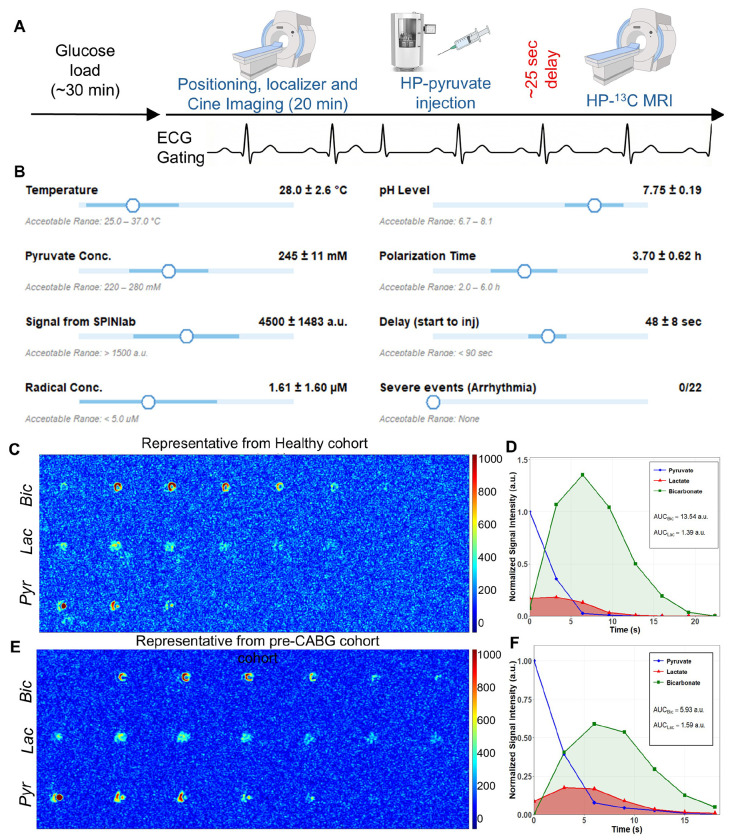
Hyperpolarized ^13^C MRI Protocol and Quality Control. **(A)** Imaging protocol timeline. Subjects underwent oral glucose loading (approximately 30 minutes) followed by positioning and cine imaging (20 minutes). Hyperpolarized [1-^13^C]pyruvate was injected intravenously with electrocardiogram (ECG)-gated ^13^C imaging commencing approximately 25 seconds post-injection. **(B)** Quality control parameters for all acquisitions demonstrating consistent polarization performance across temperature, pH, pyruvate concentration, signal intensity, dissolution-to-injection delay time, and residual radical concentration. No severe arrhythmia events were observed across all 22 injections (0/22 in 10 healthy, 6 pre-CABG and 6 Post-CABG scans). **(C–D)** Representative Healthy Volunteer: **(C)** Whole-heart dynamic spectral images showing metabolite distribution. (**D)** Time-resolved metabolite signal intensity curves extracted specifically from the myocardium (normalized to maximum pyruvate signal), demonstrating high PDH mediated oxidative activity (AUC_Bic_=13.54). **(E–F)** Representative Pre-CABG Patient: **(E)** Whole-heart dynamic spectral images. **(F)** Corresponding myocardial signal curves (normalized to maximum pyruvate) in myocardium, revealing blunted oxidative metabolism (AUC_Bic_=5.93). AUC = area under the curve; Bic = bicarbonate; Lac = lactate; Pyr = pyruvate.

**Figure 3:**
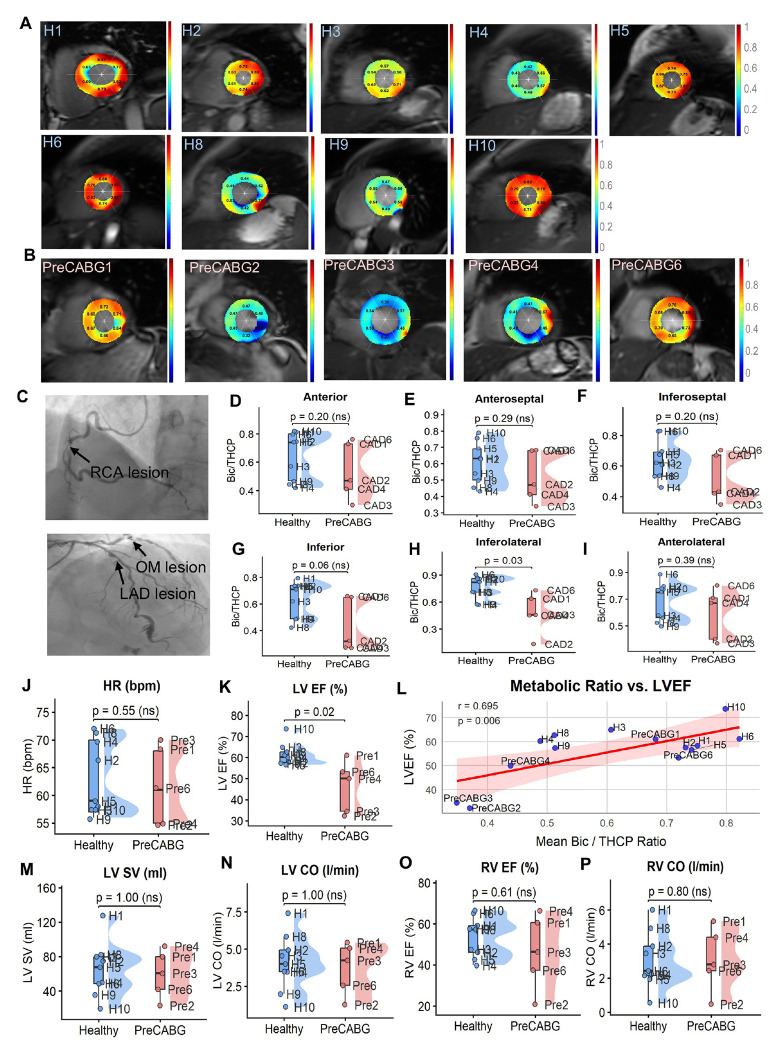
Regional Bicarbonate-to-Total Hyperpolarized Carbon Product (Bic/THCP) Ratio Maps. **(A)** Bic/THCP ratio map overlays on anatomic image for all healthy controls (H1-H10, excluding H7) demonstrating relatively uniform metabolic activity across myocardial segments. **(B)** Bic/THCP ratio maps for pre-CABG patients (Pre-CABG1-Pre-CABG6, excluding Pre-CABG5) showing regional metabolic heterogeneity with reduced bicarbonate production in specific territories. **(C)** Representative coronary angiography demonstrating stenoses in right coronary artery (RCA), left circumflex (obtuse marginal (OM)), and left anterior descending (LAD) territories. **(D-I)** Quantitative comparison of regional Bic/THCP ratios across varying segments (Anterior, Anteroseptal, Inferoseptal, Inferior, Inferolateral, Anterolateral). **(J-P)** Global hemodynamic and functional metrics comparing healthy controls and pre-CABG patients. Bic/THCP ratios were significantly lower in the inferolateral segment in pre-CABG patients compared with healthy controls (p=0.03).

**Figure 4:**
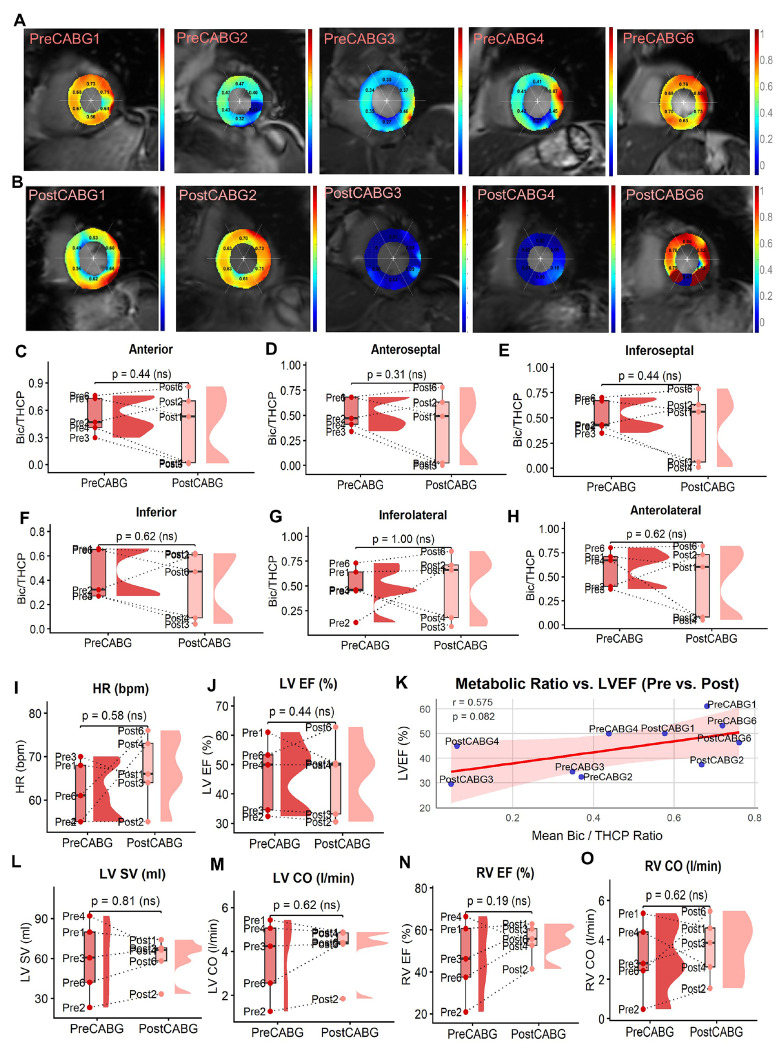
Pre-CABG Versus Post-CABG Bic/THCP Ratio Comparison. **(A)** Bic/THCP ratio map overlays on anatomic image for pre-CABG patients. **(B)** Corresponding post-CABG maps for the same patients. **(C-H)** Regional quantitative comparisons of Bic/THCP ratios before and after surgery. **(I-O)** Changes in global hemodynamic parameters following revascularization: Heart Rate **(I)**, LV Ejection Fraction **(J)**, Bic/THCP ratio versus LV EF correlation in the pre- and post-CABG cohort **(K)**, LV Stroke Volume **(L)**, LV Cardiac Output **(M)**, RV Ejection Fraction **(N)**, and RV Cardiac Output **(O)**.

**Figure 5:**
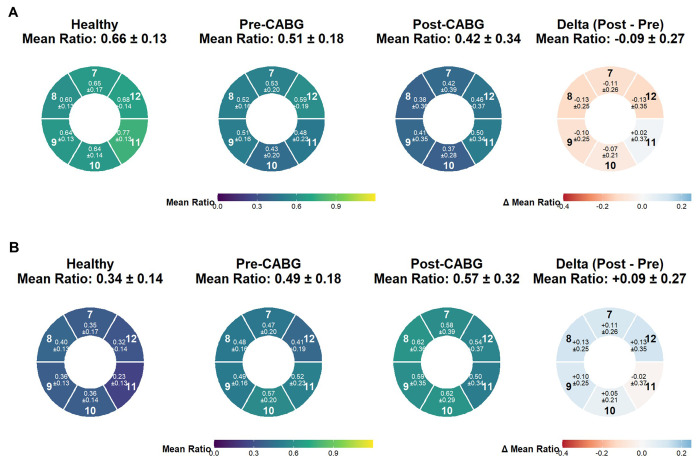
Polar Maps of Regional Metabolic Parameters. **(A)** Mean Bicarbonate-to-Total Hyperpolarized Carbon Product (Bic/THCP) ratio maps for Healthy controls, Pre-CABG, and Post-CABG patients, including quantitative difference (Δ_Post - Pre_). **(B)** Mean Lactate-to-THCP (Lac/THCP) ratio maps. Note the regional metabolic impairment (reduced Bic/THCP) localized to the inferolateral territory (AHA segment 11) in the Pre-CABG cohort.

**Figure 6:**
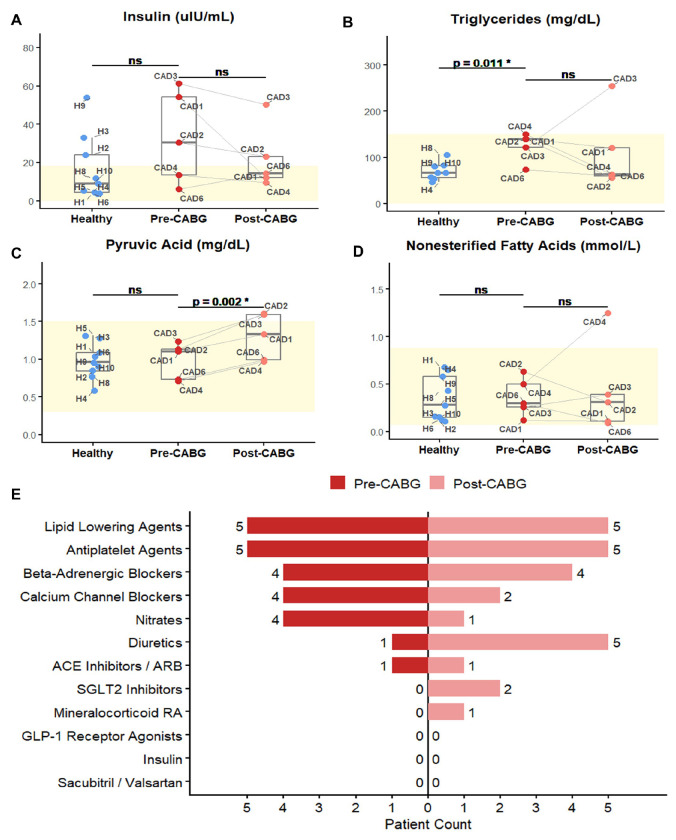
Circulating Metabolic Substrates Before and After CABG. (A–D) Serum levels for (A) Insulin, (B) Triglycerides, (C) Pyruvic Acid, and (D) Nonesterified Fatty Acids (NEFA) across Healthy controls and CAD patients (Pre- and Post-CABG). The yellow shaded region in each plot indicates the established normal reference range for the respective substrate. (E) Distribution of patient medications within the CAD cohort, comparing the number of patients on specific therapies before (dark red) and after (light red) CABG.

Clinical-grade [1-^13^C]pyruvic acid (Isotec, Sigma-Aldrich) was polarized using a SPINlab clinical polarizer (GE Healthcare, Waukesha, WI, USA) according to previously validated protocols^26,27^. Briefly, 1.47 g of neat pyruvic acid containing 15 mM trityl radical (AH111501, GE Healthcare) and 1 mM gadolinium chelate was placed in the polarization chamber and subjected to microwave irradiation at 3.35 T and 0.85 K. Following polarization (3.70 ± 0.62 hours), the sample was rapidly dissolved in superheated sterile water and neutralized to physiological pH. Rigorous quality control parameters were verified before injection, consistent with investigational new drug requirements (Supplementary Figure 1). Quality control metrics included: temperature, pH, pyruvate concentration, signal from SPINlab, time from dissolution to injection, and residual radical concentration. Injections were administered as a 0.4 mL/kg bolus (0.1 mmol/kg) over 20 seconds into an antecubital vein, followed by a 25 mL saline flush (Figure 2).

### MRI Protocol

All imaging was performed on a 3T clinical MRI scanner (750w Discovery, GE Healthcare, Waukesha, WI) equipped with a custom-built two-loop ^13^C Helmholtz surface coil (PulseTeq Limited; Chobham, Surrey, UK) for ^13^C imaging and the body coil for ^1^H localization. Subjects underwent a standardized glucose load (48 g oral glucose) approximately 30 minutes before imaging to stimulate insulin secretion and enhance myocardial glucose uptake and PDH activity. Following glucose loading, subjects were positioned in the scanner for anatomical reference imaging. Proton imaging included: balanced steadystate free precession cine imaging in standard cardiac views (short-axis stack, 2-chamber, 3-chamber, 4-chamber and long-axis stack) for volumetric analysis. The protocol used a field of view of 400 × 400 mm^2^ with a spatial resolution of 2.08 × 2.08 mm^2^ and slice thickness of 8 mm. Key sequence parameters included a flip angle of 50°, echo time (TE) of 1.2 ms, repetition time (TR) of 3.4 ms, and acquisition of 30 cardiac phases. Following completion of proton imaging, HP [1-^13^C]pyruvate was injected, and ^13^C imaging commenced ~ 25 seconds post-injection to target the left ventricular bolus arrival.

Hyperpolarized ^13^C imaging employed a slice-selective spectral-spatial excitation pulse with metabolite-specific flip angles (pyruvate 10°, lactate 90°, bicarbonate 90°) to optimize signal while preserving hyperpolarization. Dynamic acquisition parameters included: field of view (FOV): 400 × 400 mm^2^, matrix 40 x 40, data acquisition every R-R interval at end-diastole, in-plane resolution 10 mm x 10 mm, slice thickness 30 mm, and temporal resolution of 1 cardiac cycle over 60 seconds of acquisition. Electrocardiographic gating was employed to minimize cardiac motion artifacts. All subjects received at least one HP [1-^13^C]pyruvate injection for short-axis (SAX) cardiac imaging, with select subjects receiving an additional HP [1-^13^C]pyruvate injection for long-axis (LAX) imaging within the same session. All procedures for hyperpolarized pyruvate production and injection, ^1^H MRI acquisition, ^13^C MRI acquisition, and subject monitoring were conducted in accordance with previously published studies and consensus statement^26–28^.

### Image Analysis and Metabolic Quantification

Proton cine images were analyzed using commercial software (QMass, Medis Medical Imaging Systems) for quantification of left ventricular volumes, ejection fraction, and wall motion. Hyperpolarized ^13^C data were processed using MATLAB (R2025b; MathWorks, Natick, MA) based *HP-^13^CMRAnalyst* (custom software; Supplementary Figure 2) incorporating established methodologies^29^. Segmental analysis followed the American Heart Association 17-segment model ^30^. Data were analyzed in segments 7 to 12. Metabolic ratios were calculated for each myocardial segment. Total hyperpolarized carbon-13 product (THCP) was defined as the sum of hyperpolarized [1-^13^C]lactate and [^13^C]bicarbonate signals. Primary metabolic metrics included: bicarbonate-to-THCP ratio (Bic/THCP), reflecting PDH-mediated pyruvate-to-bicarbonate conversion; lactate-to-THCP ratio (Lac/THCP), reflecting lactate dehydrogenase activity; and total metabolic activity (sum of bicarbonate and lactate signals within the myocardium). While these experiments were not designed to calculate kinetic rate constants, apparent kinetic rate constants were estimated using a simplified two-site exchange model ^31,32^. This approach eliminates the need for arterial input function modeling by using the measured pyruvate signal directly as the driving function. Model fitting was performed on a voxel-wise basis using nonlinear least-squares optimization, with constraints applied to ensure physiologically plausible solutions. Additional methodological details, assumptions, and validation analyses are provided in the Supplementary Material.

### Laboratory Assessments

Fasting blood samples were obtained before each imaging session for metabolic substrate analysis. Measurements included serum glucose, insulin, triglycerides, non-esterified fatty acids (NEFA), and pyruvic acid. Comprehensive metabolic panels and complete blood counts were performed to ensure eligibility and safety (Supplementary Table 1).

### Statistical Analysis

Continuous variables are presented as mean ± standard deviation or median (interquartile range) as appropriate. Categorical variables are presented as counts and percentages. Comparisons between healthy controls and pre-CABG patients employed the Mann-Whitney U test for continuous variables and Fisher exact test for categorical variables, given the small sample sizes. Paired comparisons between pre-CABG and post-CABG measurements used the Wilcoxon signed-rank test. Correlations between metabolic parameters and ventricular function were assessed using Spearman rank correlation. Regional metabolic differences across coronary territories were evaluated using analysis of variance with post-hoc Tukey correction. A two-sided p-value≤0.05 was considered statistically significant. Given the exploratory nature of this study, analyses were focused on effect size estimation and physiological plausibility. No formal correction for multiple comparisons was applied to avoid Type II errors in this pilot cohort; results should be interpreted as hypothesis-generating. Statistical analyses were performed using R Statistical Software (v4.5.2; R Core Team, 2025) and GraphPad Prism (v10, GraphPad Software).

## Supplementary Material

This is a list of supplementary fi les associated with this preprint. Click to download.

• NatureCVRManuscriptSupplementaryMaterialFinal04282026.pdf

## Figures and Tables

**Table 1.** Baseline Demographics and Clinical Characteristics. Comparison of demographic data, comorbidities, and anthropometric measurements between Healthy Controls (n=9) and patients with Three-Vessel Coronary Artery Disease (CAD, n=5). Values are presented as mean ± standard deviation or count (percentage). BMI = body mass index; CABG = coronary artery bypass grafting; COPD = chronic obstructive pulmonary disease.

Characteristic	Healthy Controls	CAD Patients
	(n=9)	(n=5)
**Demographics**		
Age, y	36 ±4*	64 ± 12
Sex		
Male	3 (33.3)	5 (100.0)
Female	6 (66.7)	0 (0.0)
Race		
White	5 (55.6)	3 (60.0)
Black or African American	1 (11.1)	0 (0.0)
Asian	3 (33.3)	0 (0.0)
Other or Unknown	0 (0.0)	2 (40.0)†
**Ethnicity**		
Hispanic or Latino	2 (22.2)	1 (20.0)
Not Hispanic or Latino	7 (77.8)	4 (80.0)
**Clinical History & Comorbidities**		
Hypertension	—	4 (80.0)
Hyperlipidemia	—	3 (60.0)
Smoking History (Former)	—	3 (60.0)
Diabetes or Prediabetes	—	2 (40.0)
Obstructive Sleep Apnea	—	2 (40.0)
History of Malignancy	—	2 (40.0)
Obesity (BMI ≥ 30 kg/m^2^!	—	1 (20.0)
Peripheral Vascular Disease	—	1 (20.0)
COPD / Emphysema	—	1 (20.0)
Celiac Disease	—	1 (20.0)
**Anthropometry & Study Timing**		
BMI, kg/m^2^	24.2 ± 1.0*	29.1 ± 1.2
Time from Pre-CABG Imaging to Surgery, days	—	14 ± 9
Time from Surgery to Post-CABG Imaging, days	—	120 ± 23

* p < 0.05 vs CAD Patients.

## Data Availability

De-identified individual participant data and the custom MATLAB-based analysis software (HP-^13^CMRAnalyst) are available from the corresponding authors upon reasonable request, subject to institutional data sharing agreements and applicable privacy regulations.
